# Treatment with Elapegademase Restores Immunity in Infants with Adenosine Deaminase Deficient Severe Combined Immunodeficiency

**DOI:** 10.1007/s10875-024-01710-z

**Published:** 2024-04-27

**Authors:** Elizabeth Daly Hicks, Geoffrey Hall, Michael S. Hershfield, Teresa K. Tarrant, Pawan Bali, John W. Sleasman, Rebecca H. Buckley, Talal Mousallem

**Affiliations:** 1 Department of Pediatrics, Division of Pediatric Transplant and Cellular Therapies, Duke University Medical Center, Durham, NC USA; 2 Department of Pediatrics, Division of Pediatric Allergy and Immunology, Duke University Medical Center, Durham, NC USA; 3 Department of Medicine, Division of Rheumatology and Immunology, Duke University School of Medicine, Durham, NC USA; 4 Department of Biochemistry, Duke University School of Medicine, Durham, NC USA; 5 Department of Medicine, Division of Rheumatology, Durham Veteran Affairs Medical Center, Durham, NC USA; 6 Department of Pediatrics, Division of Allergy and Immunology, Duke University School of Medicine, Durham, NC USA

**Keywords:** Adenosine deaminase deficiency, Severe combined immunodeficiency, Enzyme replacement therapy, ADA-SCID, Elapegademase, Immune reconstitution

## Abstract

**Purpose:**

Patients with adenosine deaminase 1 deficient severe combined immunodeficiency (ADA-SCID) are initially treated with enzyme replacement therapy (ERT) with polyethylene glycol-modified (PEGylated) ADA while awaiting definitive treatment with hematopoietic stem cell transplant (HSCT) or gene therapy. Beginning in 1990, ERT was performed with PEGylated bovine intestinal ADA (ADAGEN®). In 2019, a PEGylated recombinant bovine ADA (Revcovi®) replaced ADAGEN following studies in older patients previously treated with ADAGEN for many years. There are limited longitudinal data on ERT-naïve newborns treated with Revcovi.

**Methods:**

We report our clinical experience with Revcovi as initial bridge therapy in three newly diagnosed infants with ADA-SCID, along with comprehensive biochemical and immunologic data.

**Results:**

Revcovi was initiated at twice weekly dosing (0.2 mg/kg intramuscularly), and monitored by following plasma ADA activity and the concentration of total deoxyadenosine nucleotides (dAXP) in erythrocytes. All patients rapidly achieved a biochemically effective level of plasma ADA activity, and red cell dAXP were eliminated within 2–3 months. Two patients reconstituted B-cells and NK-cells within the first month of ERT, followed by naive T-cells one month later. The third patient reconstituted all lymphocyte subsets within the first month of ERT. One patient experienced declining lymphocyte counts with improvement following Revcovi dose escalation. Two patients developed early, self-resolving thrombocytosis, but no thromboembolic events occurred.

**Conclusion:**

Revcovi was safe and effective as initial therapy to restore immune function in these newly diagnosed infants with ADA-SCID, however, time course and degree of reconstitution varied. Revcovi dose may need to be optimized based on immune reconstitution, clinical status, and biochemical data.

## Introduction

Adenosine deaminase 1 (ADA) deficiency is an autosomal recessive disorder of purine metabolism resulting in elevated levels of adenosine (Ado) and deoxyadenosine (dAdo) in plasma and urine [1, 2]. The accumulation of intracellular dAdo nucleotides (dAXP) causes lymphocyte apoptosis and lymphopenia [1, 2]. A majority of affected patients have a near complete loss of ADA function resulting in a profound depletion of T-cells, B-cells, and NK-cells; however about 20% of cases have a less complete deficiency of ADA and can present with delayed onset of combined immunodeficiency (CID) [1, 3].

Currently, management consensus is that newly diagnosed patients should initially receive enzyme replacement therapy (ERT) with polyethylene glycol-modified (PEGylated) ADA [3]. Starting ERT early may reverse metabolic toxicity to the thymus and other organs, thus stabilizing patients prior to definitive treatment. While ERT may be lifesaving, it does not provide full, long-lasting immune reconstitution; therefore, patients still require definitive therapy with either allogeneic hematopoietic stem cell transplantation (HSCT) or autologous hematopoietic stem cell gene therapy [3].

Following the successful treatment of two infants with ADA-SCID using PEGylated bovine intestinal ADA (ADAGEN®) at Duke University Medical Center in the 1980s [4], ADAGEN became the first PEGylated protein and first form of ERT to be approved by the United States Food and Drug Administration (FDA). However, sourcing and safety concerns related to the use of purified bovine proteins led to the development of Revcovi®, a PEGylated recombinant bovine ADA for ERT. In 2019, the use of ADAGEN was discontinued, and Revcovi became the only available form of ERT for ADA deficiency. The clinical trials that led to FDA approval of Revcovi primarily included adults and children who were previously treated for many years with ADAGEN [5]. There are limited data on the use of Revcovi to treat ERT-naïve infants with ADA-SCID [6]. We report our experience in managing three ADAGEN-naïve infants with newly diagnosed ADA-SCID using Revcovi as initial therapy.

## Methods

The legal representatives for all participants provided written consent for *enrollment into protocol, Genetic and Functional Analysis of Primary Immune Deficiencies* (Pro00066839) which was approved by the institutional review board of Duke University.

## Results

### Clinical Presentation: Case 1

Patient 1 is a White male, born to non-consanguineous parents, who had an abnormal newborn screen (NBS) for SCID. Initial flow cytometry at 9 days of life showed 1 CD3^+^ cell/µL, 5 CD19^+^ cells/µL, and 50 CD16/56^+^ NK-cells/µL. Lymphocyte proliferation in response to phytohemagglutinin (PHA) stimulation was unsuccessful prior to starting ERT due to profound lymphopenia. However, after 4 weeks of ERT the proliferation was detectable, but low, at 22,313 counts/minute (cpm) (< 5% normal). Engraftment of maternal T-cells was not detected. Erythrocyte ADA activity was 0.2 nmol/h/mg protein (normal range 63.0 ± 41.4) and dAXP was markedly elevated at 0.62 µmol/mL of packed red blood cells (pRBCs), or 61.7% of total adenine nucleotides (normal range < 0.002, < 0.2%). Genetic testing confirmed ADA deficiency with a homozygous pathogenic variant in *ADA1* [c.956_960del (p.Glu319Glyfs*3)]. Subcutaneous immunoglobulin replacement (SCIG) and antimicrobial prophylaxis with trimethoprim-sulfamethoxazole and fluconazole were started. At 30 days of life, Revcovi was started with an initial dose of 0.2 mg/kg twice weekly, as per manufacturer’s instructions.

The patient developed immune reconstitution of T-cells, B-cells and NK-cells and was able to discontinue antimicrobial prophylaxis and SCIG at around 17 and 37 weeks of ERT, respectively. Revcovi dosing was consolidated to once weekly and the initial total dose remained unchanged as he gained weight. Plasma ADA activity and dAXP levels in pRBCs were measured regularly to ensure appropriate dosing. Between weeks 61 and 67 of therapy, lymphocyte counts and function had fallen despite adequate ADA activity (> 30µmol/h/mL) and detoxification (dAXP undetectable). Due to progressive lymphopenia, declining T-cell function and hypogammaglobulinemia, both SCIG and antimicrobial prophylaxis were restarted. Additionally, the weight-based dose of Revcovi had fallen to 0.16 mg/kg once weekly, so the dose was increased to 0.125 mg/kg twice weekly during week 63. The adjustment of Revcovi dose and frequency re-established normal T-cell function prompting discontinuation of SCIG and antimicrobial prophylaxis. His T- cell counts have remained low, which has previously been noted among patients treated with ERT (ADAGEN) after a peak around 1–3 years [7]. Despite low T-cell counts the patient remained free of significant infections throughout his clinical course. He developed IgE-mediated food allergy to egg. He is currently at the 67th percentile for length and 19th percentile for weight, with a weight-for-length ratio of ~ 3rd percentile that has been consistent over time. He has speech delay with no evidence of hearing loss.

### Clinical Presentation: Case 2

Patient 2 is a Black male, born to non-consanguineous parents, who had an abnormal NBS for SCID. Immune evaluation showed 159 CD3^+^ cells/µL, 27 CD19^+^ cells/µL, 207 CD16/56^+^ NK-cells/µL, and 11 naïve CD4^+^ cells/µL. Lymphocyte proliferation in response to PHA stimulation was detectable, but low, at 17,018 cpm (< 5% normal). Engraftment of maternal T-cells was not detected. Erythrocyte ADA activity was undetectable and dAXP was markedly elevated at 0.61 µmol/mL of pRBCs, or 34.8% of total adenine nucleotides. Genetic testing showed 2 pathogenic heterozygous variants in *ADA1* [c.632G > A (p.Arg211His) and c.845G > A (p.Arg282Gln)] consistent with ADA deficiency. SCIG and antimicrobial prophylaxis were started. He was hospitalized for pneumonia at 5 weeks of life, with suspicion for pulmonary alveolar proteinosis. He had subsequent resolution of symptoms following initiation of Revcovi (at 7 weeks of life) using 0.2 mg/kg twice weekly [8].

The patient showed progressive increase in lymphocyte counts after starting ERT. Antimicrobial prophylaxis was discontinued at around 18 weeks of therapy after normalization of T-cell function and development of newly formed naïve T-cells in the circulation. SCIG was stopped after 39 weeks of ERT. The dose of Revcovi was increased after 11 weeks of ERT, and the total dose remained unchanged while gaining weight as he continued to show adequate ADA activity and near undetectable dAXP. Now, two years old, he continues to be free of infections while on a Revcovi dose, unadjusted for weight gain due to adequate clinical and biochemical response, of 0.1 mg/kg twice weekly. He is developing normally with most recent growth at the 13th percentile for weight and 32nd percentile for length.

### Clinical Presentation: Case 3

Patient 3 is a White female, born to non-consanguineous parents, who had two borderline NBSs for SCID. Flow cytometry at 50 days of life showed lymphopenia with 192 CD3^+^ cells/µL, 9 CD19^+^ cells/µL, and 22 CD16/56^+^ cells/µL. CD3^+^ count had decreased to 169 cells/µL by the time of referral to our institution at 71 days of life. Lymphocyte proliferation in response to PHA stimulation was detectable, but low, at 10,242 cpm (< 5% normal). Engraftment of maternal T-cells was not detected. Erythrocyte ADA activity was absent with increased dAXP concentration at 0.923 µmol/mL of pRBCs, or 40.3% of total adenine nucleotides. Genetic testing revealed two heterozygous variants in *ADA1* [c.445 C > T (p.Arg149Trp) and c.956_960del (p.Glu319Glyfs*3)] consistent with ADA deficiency. Antimicrobial prophylaxis with trimethoprim-sulfamethoxazole and fluconazole were started. At 84 days of life Revcovi was started at 0.2 mg/kg twice weekly, as well as SCIG. At 7 months of ERT, the dose and frequency of Revcovi was consolidated to 0.3 mg/kg once weekly.

The patient demonstrated earlier than expected immune reconstitution after 3 weeks of ERT with normalization of T-cell, B-cell, and NK-cell counts and normal proliferation to PHA. Plasma ADA activity at this time was 109.4 µmol/h/mL with 0.214 µmol/mL dAXP concentration in pRBCs (9.7% of total adenine nucleotides). Immunoglobulin replacement was stopped after 8 weeks of ERT and antimicrobial prophylaxis was stopped around 4 months of ERT.

The patient had mild transient transaminitis prior to starting ERT that resolved. She is 17 months old and has been on ERT for 15 months at the time of this report. She is doing well with no infections. She is growing and developing normally with current weight at the 79th percentile and length at the 19th percentile.

### Biochemical Studies

**Fig. 1:**
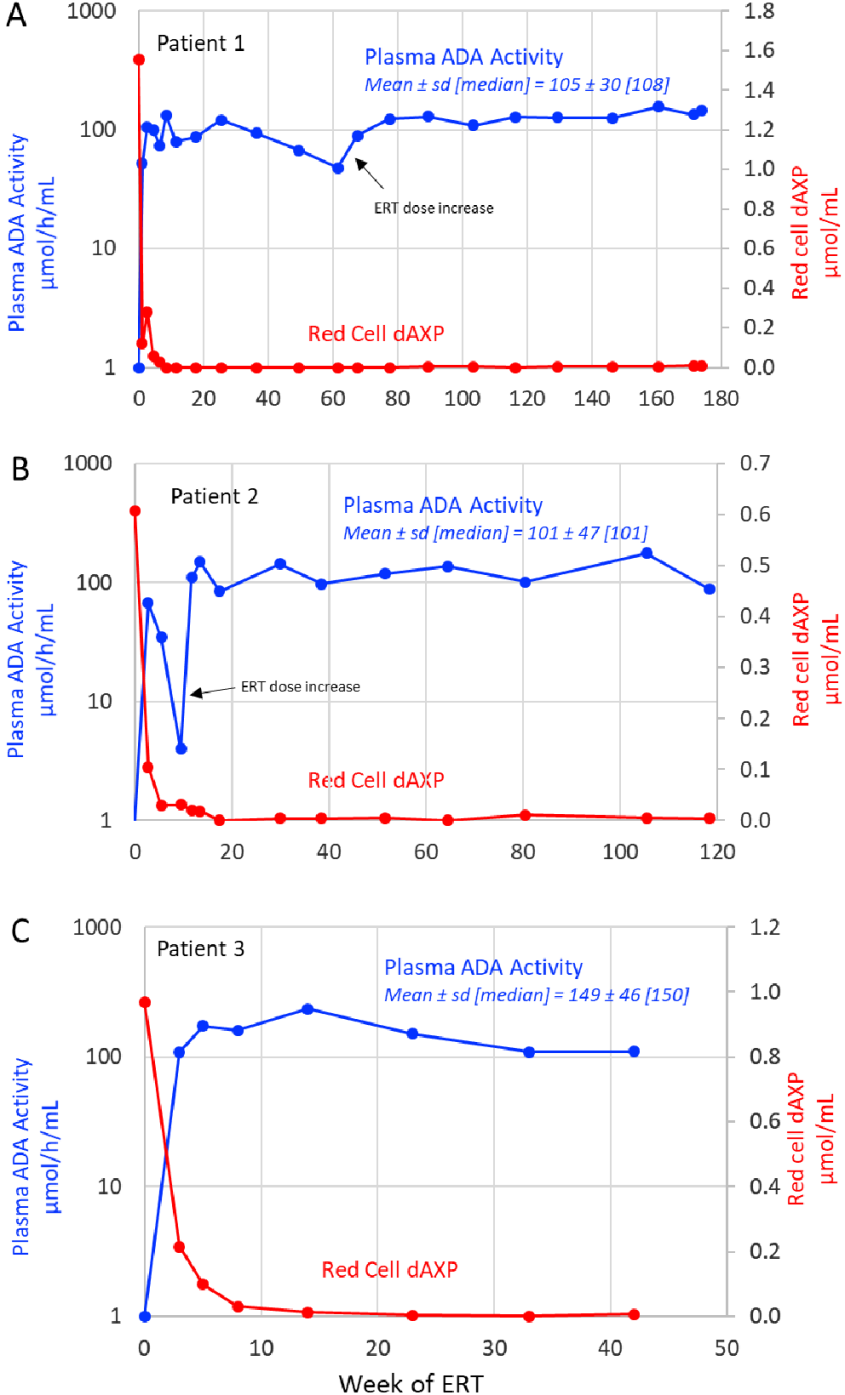
**Biochemical Studies**. The plasma ADA activity and pRBC dAXP concentration for patient 1 **A**, patient 2 **B** and patient 3 **C**. All patients had a high dAXP concentration prior to initiating therapy and achieved rapid detoxification and maintained a dAXP concentration below 0.01 µmol/mL throughout treatment. All patients maintained ADA activity well above the goal of 30 µmol/h/mL. Patient 1’s ADA activity gradually fell from 120 to 48 µmol/h/mL, but it increased again following Revcovi dose escalation. Patient 2 had a brief drop below goal at week 9 likely related to home-dosing error, but remained above goal thereafter

Prior to initiating ERT, all three patients had near absent ADA activity in pRBCs and markedly elevated levels of erythrocyte dAXP, accounting for 40–60% of total deoxyadenosine nucleotides (dAXP are normally undetectable in mature red cells). A trough plasma ADA activity above 30 µmol/h/mL (normally < 0.5 µmol/h/ml in healthy individuals) is recommended by the manufacturer in order to reduce pRBC dAXP to less than 0.01 µmol/ml [9]. The plasma ADA threshold was rapidly achieved in all three patients, with median levels exceeding 3–5 fold the suggested trough while on Revcovi (Fig. 1A-C). In patient 2, plasma ADA activity declined transiently to 4 µmol/h/ml at week 9 owing to suspected incorrect home administration, but returned to effective levels after the recommended dose of Revcovi was restored at week 11 (Fig. 1B). In patients 1 and 3, red cell dAXP declined steadily, becoming negligible by week 8 of treatment in patient 1 and between weeks 8 and 14 in patient 3 (Fig. 1A & C). Elimination of dAXP in patient 2 was delayed until week 17 owing to the transient decline in plasma ADA activity (Fig. 1B).

### Immune Reconstitution

**Fig. 2:**
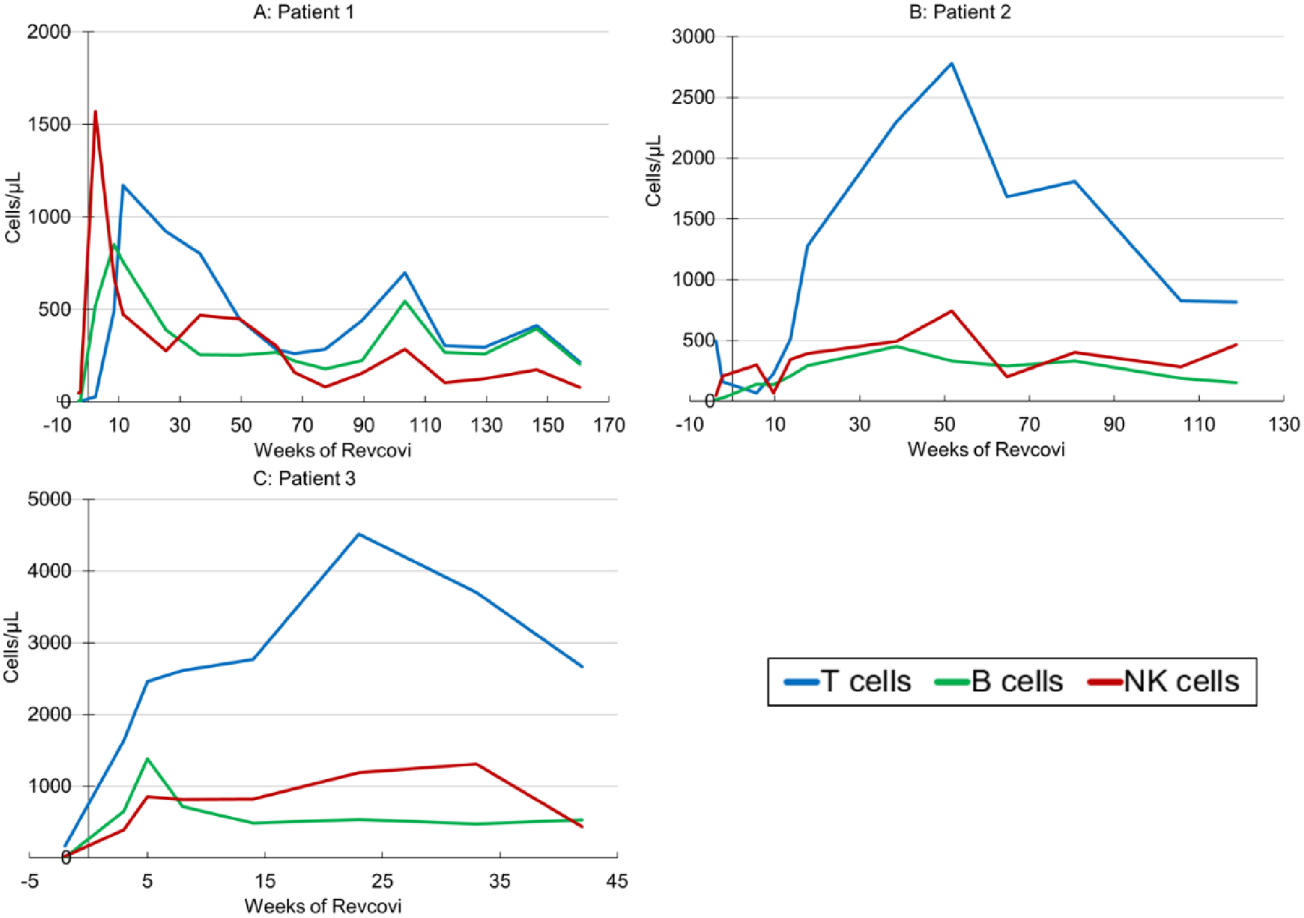
**Immunophenotyping**. Lymphocyte counts for patient 1 **A**, patient 2 **B**, and patient 3 **C.** Patients 1 & 2 showed improvement with reconstitution at around 2 months of ERT, whereas patient 3 developed full immune reconstitution at 3 weeks. *T-cells: CD3*^*+*^, *B-cells: CD19*^*+*^, *NK-cells: CD16/56*^*+*^

After initiating ERT, lymphocyte counts rapidly improved in all patients. An increase was seen first in NK-cell and B-cell subsets, followed by T-cells in the first 2 patients. Unexpectedly, patient 3 had normalization of T-cells (including naïve) by the third week of ERT, while it took longer to achieve T-cell reconstitution in patients 1 and 2 (Fig. 2A-C). NK-cell numbers normalized within 3–5 weeks of therapy for all patients. B-cells normalized by 3 weeks in patients 1 and 3. In patient 2, B-cells increased steadily after starting therapy, and are now in the normal range.

**Fig. 3:**
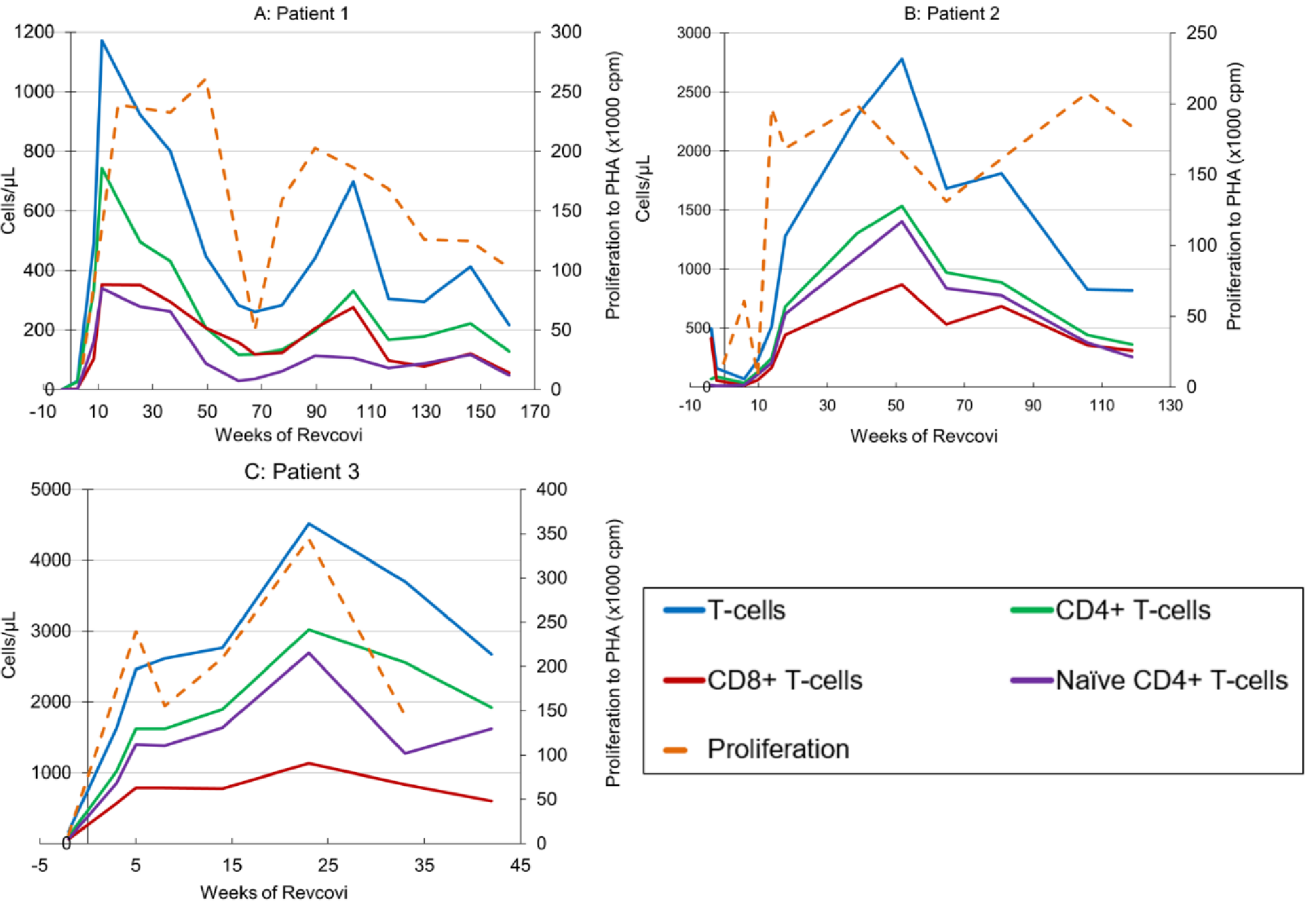
**T-cell Subsets and Mitogen Proliferation**. **A** Patient 1’s T-cell numbers and mitogen proliferation. T-cell function declined over the first year, but then increased again after an increase in dose of ERT. **B** Patient 2’s T-cells numbers and mitogen proliferation have improved since starting ERT and are now normal. **C** Patient 3’s T-cell function and counts have normalized since week 3 of ERT. *Naïve T-cells: CD4/CD45RA/CD62L*^*+*^

Naïve T-cells (CD4/CD45RA/CD62L^+^) first appeared at week 8 of therapy in patient 1, week 9 in patient 2 and week 3 in patient 3. T-cell function, as measured by proliferation in response to PHA, normalized by week 11 in patient 1, week 13 in patient 2 and by week 5 in patient 3. T-cell reconstitution was evident 11 weeks into ERT for patient 1, with progressive improvement over time, however, by week 67 all subsets subsequently declined. T-cells had decreased to 262 cells/µL, and T-cell function was also depressed, despite adequate ADA activity and undetectable pRBC dAXP. The Revcovi dose was increased at this time from 0.16 mg/kg weekly to 0.13 mg/kg, twice weekly. He demonstrated a favorable response to this dose increase, with normalization of T-cell function. After 2 years of Revcovi his T-cell count was 699 cells/µL while receiving 0.20 mg/kg divided twice weekly. However, recently T-cell counts have begun to decline (218 CD3^+^ cells/µL) despite appropriate ADA plasma activity and undetectable dAXP levels. Reassuringly, T-cell function remains normal. In patient 2, the lymphocyte subsets remain sufficient with normal T-cell function. In patient 3, all lymphocyte subsets remain completely normal and T-cell function remains intact (Fig. 3 C). Lymphocyte subsets for all patients by weeks of ERT can be found in Table 1.

**Table 1:** Lymphocyte subsets by week of ERT for all patients. Refer reader to Shearer et. al. for lymphocyte reference ranges [17]

Subset			Cells/µL (%) by Weeks on Revcovi									
**Patient 1**	**(-) 3**	**2**	**8**	**11**	**25**	**49**	**67**	**89**	**103**	**129**	**160**	
ALC	52 (2%)	2592 (24%)	2055 (47.8%)	2520 (60%)	1610 (46%)	1166 (22%)	570 (19%)	850 (25%)	1564 (34%)	704 (16%)	528 (12%)	
CD3^+^	1 (2.6%)	29 (1.1%)	493 (24.0%)	1172 (46.5%)	923 (57.3%)	448 (38.4%)	285 (50.0%)	442 (52.0%)	699 (44.7%)	296 (42.1%)	218 (41.3%)	
CD4^+^	2 (3.0%)	26 (1.0%)	333 (16.2%)	743 (29.5%)	496 (30.8%)	208 (17.8%)	134 (23.5%)	198 (233%)	332 (21.2%)	179 (25.4%)	130 (24.6%)	
CD8^+^	0 (0.1%)	3 (0.1%)	105 (5.1%)	353 (14.0%)	351 (21.8%)	208 (17.8%)	125 (21.9%)	206 (24.2%)	277 (17.7%)	79 (11.2%)	58 (11.0%)	
CD19^+^	54 (9.6%)	524 (20.2%)	851 (41.4%)	759 (30.1%)	391 (24.3%)	253 (21.7%)	180 (31.5%)	225 (26.5%)	546 (34.9%)	261 (37.1%)	206 (39.0%)	
NK-cells (CD16^+^/56^+^)	50 (96.7%)	1571 (60.6%)	676 (32.9%)	476 (18.9%)	277 (17.2%)	449 (38.5%)	82 (14.3%)	157 (18.6%)	285 (18.2%)	127 (18.1%)	80 (15.1%)	
CD3^+^/CD45RA^+^	NA	NA	313 (63.5%)	892 (76.1%)	654 (70.9%)	272 (60.8%)	163 (57.2%)	255 (57.8%)	410 (58.6%)	109 (36.7%)	97 (44.4%)	
CD3^+^/CD45RO^+^	NA	NA	87 (17.6%)	98 (8.4%)	79 (8.6%)	68 (15.2%)	38 (13.4%)	67 (15.2%)	127 (18.1%)	95 (32.2%)	71 (32.5%)	
Naïve T-cells (CD4^+^/CD45RA^+^/CD62L^+^)	NA	NA	162 (48.7%)	340 (45.7%)	280 (56.4%)	89 (43.0%)	63 (46.8%)	114 (57.6%)	107 (32.2%)	89 (49.8%)	49 (37.5%)	
**Patient 2**	**(-) 4**	**(-) 2**	**6**	**10**	**14**	**39**	**52**	**65**	**81**	**106**	**119**	
ALC	572 (14.3%)	408 (12%)	528 (16%)	432 (27%)	1102 (38%)	3266 (71%)	3906 (63%)	2244 (51%)	2668 (58%)	1330 (35%)		
CD3^+^	497 (86.9%)	159 (38.9%)	70 (13.3%)	228 (52.7%)	514 (46.6%)	2303 (70.5%)	2781 (71.2%)	1683 (75.0%)	1809 (67.8%)	826 (62.1%)	819 (52.0%)	
CD4^+^	69 (12.0%)	88 (21.6%)	34 (6.4%)	130 (30.2%)	247 (22.4%)	1303 (39.9%)	1535 (39.3%)	972 (43.3%)	886 (33.2%)	442 (33.2%)	362 (23.0%)	
CD8^+^	415 (72.6%)	58 (14.1%)	12 (2.2%)	59 (13.7%)	168 (15.2%)	722 (22.1%)	867 (22.2%)	534 (23.8%)	686 (25.7%)	356 (26.8%)	312 (19.8%)	
CD19^+^	14 (2.5%)	27 (6.6%)	142 (26.8%)	137 (31.7%)	209 (19.0%)	451 (13.8%)	332 (8.5%)	289 (12.9%)	331 (12.4%)	188 (14.1%)	154 (9.8%)	
NK-cells (CD16^+^/56^+^)	46 (8.1%)	207 (50.8%)	299 (56.6%)	69 (16.0%)	344 (31.2%)	493 (15.1%)	742 (19.0%)	202 (9.0%)	403 (15.1%)	283 (21.3%)	463 (29.4%)	
CD3^+^/CD45RA^+^	353 (71.0%)	64 (40.4%)	36 (50.8%)	148 (65.2%)	430 (83.8%)	2040 (88.6%)	2456 (88.3%)	1387 (82.4%)	1487 (82.2%)	625 (75.7%)	643 (78.5%)	
CD3^+^/CD45RO^+^	29 (5.8%)	47 (29.5%)	17 (24.1%)	20 (8.7%)	33 (6.4%)	106 (4.6%)	81 (2.9%)	74 (4.4%)	72 (4.0%)	88 (10.6%)	93 (11.4%)	
Naïve T-cells (CD4^+^/CD45RA^+^/CD62L^+^)	17 (24.2%)	11 (12.5%)	15 (44.4%)	108 (82.4%)	207 (83.9%)	1104 (84.7%)	1402 (91.3%)	836 (86%)	777 (87.7%)	376 (85.2%)	257 (70.9%)	
**Patient 3**	**(-) 2**	**3**	**5**	**8**	**14**	**23**	**33**	**42**	-----	-----	-----	
ALC	210 (10%)	2788 (34%)	4863 (52%)	4268 (44%)	4200 (60%)	6363 (63%)	5626 (58.0%)	3722 (46.0%)	-----	-----	-----	
CD3^+^	169 (80.4%)	1637 (58.7%)	2462 (50.9%)	2616 (61.3%)	2768 (65.9%)	4518 (71.0%)	3702 (65.8%)	2671 (70.8%)	-----	-----	-----	
CD4^+^	101 (48.0%)	1023 (36.7%)	1625 (33.6%)	1622 (38.0%)	1894 (45.1%)	3022 (47.5%)	2560 (45.5%)	1920 (50.9%)	-----	-----	-----	
CD8^+^	64 (30.3%)	572 (20.5%)	788 (16.3%)	790 (18.5%)	781 (18.6%)	1133 (17.8%)	838 (14.9%)	604 (16.0%)	-----	-----	-----	
CD19^+^	6 (2.7%)	652 (23.4%)	1383 (28.6%)	717 (16.8%)	487 (11.6%)	534 (8.4%)	473 (8.4%)	528 (14.0%)	-----	-----	-----	
NK-cells (CD16^+^/56^+^)	26 (12.5%)	390 (14.0%)	856 (17.7%)	815 (19.1%)	823 (19.6%)	1190 (18.7%)	1311 (23.3%)	438 (11.6%)	-----	-----	-----	
CD3^+^/CD45RA^+^	107 (63.2%)	1239 (75.7%)	1962 (79.7%)	2012 (76.9%)	2259 (81.6%)	3650 (80.8%)	2925 (79.0%)	2136 (80.0%)	-----	-----	-----	
CD3^+^/CD45RO^+^	21 (12.6%)	164 (10.0%)	187 (7.6%)	186 (7.1%)	125 (4.5%)	267 (5.9%)	322 (8.7%)	240 (9.0%)	-----	-----	-----	
Naïve T-cells (CD4^+^/CD45RA^+^/CD62L^+^)	69 (68.3%)	856 (83.7%)	1401 (86.2%)	1383 (85.3%)	1642 (86.7%)	2693 (89.1%)	1275 (49.8%)	1624 (84.6%)	-----	-----	-----	

**Fig. 4:**
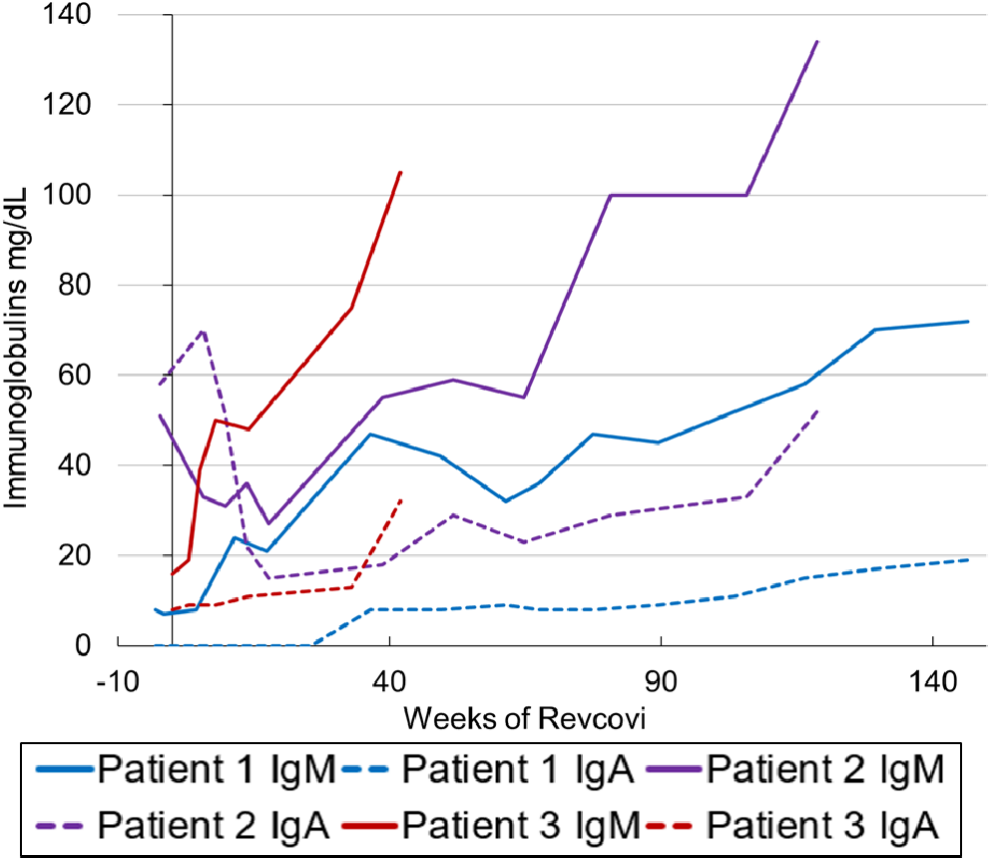
**Humoral Immunity**. All patients show evidence of endogenous immunoglobulin production and B-cell class switching with rising IgM and IgA levels

Humoral immune function normalized with Revcovi. All patients showed evidence of endogenous immunoglobulin production (rising IgM) and B-cell class switching (rising IgA levels) (Fig. 4). IgG levels measured more than 6 months from the most recent SCIG infusion were 276 mg/dL, 703 mg/dL, and 300 mg/dL for patients 1, 2 and 3, respectively. No patient has developed antibodies to Revcovi. All patients received age appropriate non-live pediatric vaccines and demonstrated normal humoral immune responses.

### Thrombocytosis

**Fig. 5:**
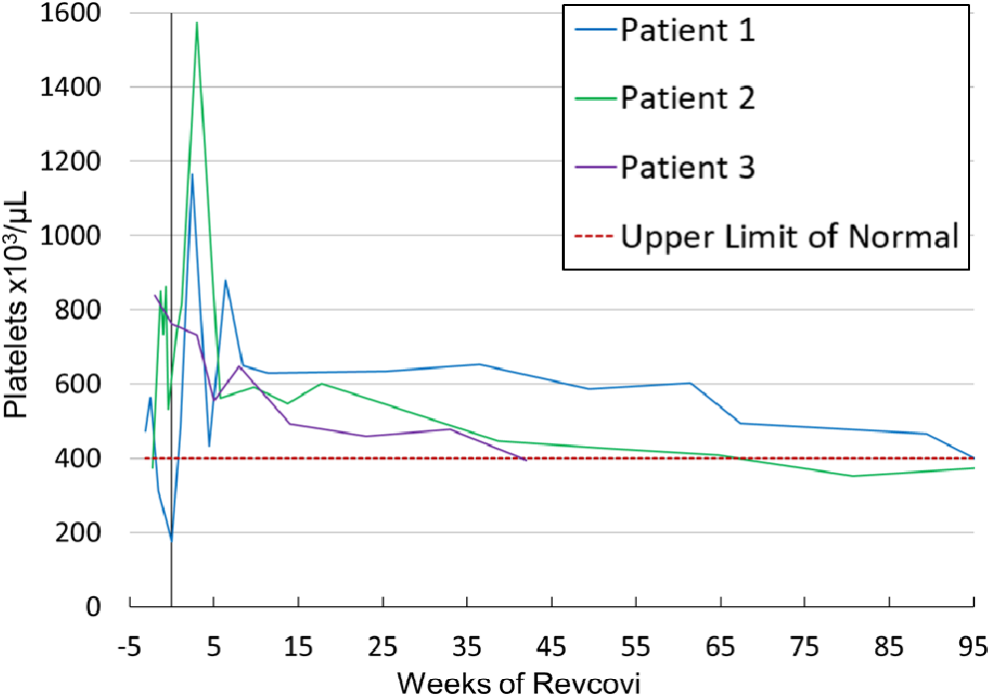
**Thrombocytosis**. Patients 1 & 2 experienced transient profound thrombocytosis in the first few weeks of therapy. Thrombocytosis quickly resolved, and platelet counts continue to be at the upper limit of normal

Patients 1 and 2 experienced significant, but transient thrombocytosis soon after starting Revcovi (Fig. 5). Patient 1’s platelet count peaked 2.5 weeks into ERT at 1,165,000/µL. Patient 2’s platelet count peaked 3 weeks into ERT at 1,573,000/µL. Patient 3 had thrombocytosis prior to starting ERT. The platelet counts in all patients decreased over the course of ERT and have nearly normalized with most recent values being around 400,000/µL. The patients experienced no thromboembolic events. ERT was not altered due to the thrombocytosis, and no anticoagulation treatment was given.

## Discussion

During the 3 decades it was available (1990–2019), ADAGEN was used as ERT in more than 300 patients with ADA-SCID (MSH unpublished work). The response to ADAGEN has been documented in numerous case reports and reviews. The literature on Revcovi is currently limited, particularly with respect to patients who had never received ADAGEN [5, 6]. We report three such ADAGEN-naïve infants with newly diagnosed ADA-SCID who were started on Revcovi within the first 1–3 months of life.

ERT should be initiated immediately following diagnosis of ADA-SCID, ideally within the first month of life. However, diagnosis can be complicated by delays in access to immunology, biochemical testing and confirmatory genetic sequencing. Additionally, financial barriers and acquiring insurance approval can prolong time to treatment with this relatively expensive therapy. For patients 2 and 3 within our cohort, there were notable delays in starting ERT. Patient 2 was hospitalized for pneumonia and Revcovi was not available on hospital formulary resulting in a delay of first dose administration [8]. Patient 3 was delayed in the diagnosis of ADA-SCID and starting ERT given the borderline TREC newborn screen and late referral to our medical center. Newborn screen results in North Carolina have 3 possible outcomes based on total quantifiable T-cell receptor excision circles (TRECs) – normal, borderline, or abnormal. Abnormal results occur with very low (below a critical threshold) or undetectable TREC counts. Borderline results occur with TREC count above the critical threshold, but below those considered a normal. The borderline result in patient 3 may be related to the presence of some T-cells initially, and a slightly higher naïve T-cell percentage than typically seen in SCID. Although the mutations were severe, genotype-phenotype variation in clinical presentation has been reported in SCID as well as other genetic conditions.

Following the initiation of ERT, plasma ADA activity rose rapidly to biochemically effective levels, resulting in the reduction of markedly elevated erythrocyte dAXP to near undetectable levels. The Revcovi package insert advises that trough plasma ADA activity be maintained above 30 µmol/h/mL [9]. However, an upper limit for plasma ADA activity has not been established in a clinical trial. In the 3 patients, the mean and median trough plasma ADA activity ranged from 100 to 165 µmol/mL. These levels are 1.5 to 2.5-fold higher than trough levels reported in patients who received comparable twice-weekly doses of ADAGEN [10, 11], as well as in the 6 older patients treated with Revcovi in the US clinical trial of Revcovi [5]. The higher levels of plasma ADA activity in the present patients were well tolerated and were not associated with adverse reactions, including hemolytic anemia, thrombocytopenia or lymphoproliferation (as was reported in post-marketing experiences with ADAGEN, and may represent intrinsic complications of ADA-SCID and not ERT). None of the 3 patients developed antibodies to Revcovi, targeting either recombinant bovine ADA or the PEG polymer.

Patients 1 and 2 have low numbers of circulating T cells, similar to what was seen in most patients receiving ERT with ADAGEN [3]. All patients remained free of significant infections with relatively appropriate growth and development, other than a speech delay in patient 1. There is no evidence of liver, lung, or skeletal abnormalities in any patient. None of the patients exhibited any symptoms of autoimmunity nor immune dysregulation, other than IgE-mediated food allergy to egg in patient 1 associated with B-cell reconstitution. While development of food allergy is likely related to an underlying genetic or environmental risk, we cannot exclude the possibility that it may signify immune dysregulation which can occur in patients with ADA deficiency.

Despite the significant immunologic reconstitution and minimal clinical adverse effects in our patients, it is important to highlight the profound thrombocytosis noted early in therapy for patients 1 and 2. Thrombocytosis has been reported in a handful of patients treated with ADAGEN and has been reported in post-marketing surveillance of Revcovi [2, 9, 12, 13]. The mechanism underlying the development of thrombocytosis in this setting is ill-defined. It may reflect bone marrow reactivity with robust hematopoiesis during immune reconstitution, release of platelets from the bone marrow, and/or increased survival time of platelets in the peripheral blood. Reassuringly, neither of our patients, nor those previously treated with ADAGEN therapy, have suffered any adverse sequelae related to thrombocytosis. It appears to be self-limited without the need for intervention or discontinuation of the ERT. Ongoing surveillance of patients treated with Revcovi will be important in identifying the frequency and severity of transient thrombocytosis compared to what was previously seen with ADAGEN.

The lymphocyte counts in patient 1, which stopped improving at around 25 weeks of ERT and continued to decline despite maintaining adequate plasma ADA activity and dAXP detoxification in red cells, are noteworthy. As shown with ADAGEN, PEGylated ADA does not enter hematopoietic cells of ADA deficient patients [4], but acts by eliminating extracellular dAdo, which is the precursor of intracellular dAXP pool expansion. Thus, plasma ADA activity and erythrocyte dAXP concentration are relevant as indicators of biochemically adequate ADA replacement with Revcovi. However, peripheral elimination of erythrocyte dAXP due to the action of PEG-ADA in plasma, may not accurately reflect the immunologic metabolic environment in the thymus, bone marrow and other lymphoid organs. The prescribing information for Revcovi recommends that plasma ADA activity should be kept above 30 µmol/h/mL [9], but pharmacokinetic data related to Revcovi dosing/frequency are limited. Despite maintaining patient 1’s mean plasma ADA activity significantly above the recommended minimum level throughout treatment, he still experienced a decline in lymphocyte counts that were congruent with a decrease in plasma ADA activity (from 80 to 100 µmol/h/mL) to 47 µmol/h/mL. In addition, the T-cell function, as evidenced by proliferation to PHA mitogen, decreased following plasma ADA activity nadir. Decreasing patient 1’s dosing frequency from twice weekly to once weekly may have also contributed to his decline in lymphocyte numbers and mitogen responses. Following an increase in his Revcovi dose and frequency, his plasma ADA activity returned to levels > 100 µmol/h/mL with subsequent improvement in T-cell function. These observations indicate the need for further systematic investigation to determine the optimal level of circulating plasma ADA activity in patients receiving Revcovi. At around week 160 of ERT the patient’s T-cell (CD3^+^) counts are 218 cells/µL. We have no plans to further escalate dosing. Prior studies of patients treated with ADAGEN showed T-cell numbers peak within 1–3 years of therapy, and this is often followed by progressive decline [7]. There is no published evidence that increasing ADAGEN dose improves immune function, nor has this been addressed in clinical trials. In cases of waning immune function while receiving ERT, more published clinical data are needed to understand immune responses to dose-escalation.

There are several pitfalls with long-term ERT including: suboptimal immune reconstitution, malignancy, immune dysregulation, the potential for developing anti-drug neutralizing antibodies, and waning lymphocyte function [1, 3, 7]. Based on our limited experience, ERT with Revcovi permits effective reconstitution of immunity and prevention of infections in newly diagnosed infants with SCID due to ADA deficiency. However, it is generally recommended that ERT not be used indefinitely, but rather as a bridge to definitive therapy with either autologous ex vivo lentiviral gene therapy or allogeneic HSCT [3, 14–16] While treatment of ADA-SCID with gene therapy showed a highly promising outcome in the United States and United Kingdom, it is currently only available on a clinical trial research basis in the United States [16]. Gene therapy marketed under brand name Strimvelis® (retroviral vector) has been approved and is available in some European countries. All patients within our cohort lack matched-related donors and are currently on the wait list for gene therapy. Proceeding with matched-unrelated HSCT remains an on-going discussion influenced by infectious complications, immunologic assessments and shared-decision making. Thus far, the patients have not experienced significant infectious complications while on ERT replacement, and have opted to postpone pursuing HSCT until clinically necessary.

## Conclusions

Our three-patient experience suggests that Revcovi provides adequate plasma ADA activity levels and maintains effective metabolic detoxification in newly diagnosed patients with ADA-SCID. Furthermore, it effectively restores immunity and prevents serious infections, which is critical to patient survival in the period before definitive therapy can be obtained. Our unique experience with the rapid immune recovery in patient 3 also highlights the importance of continued patient outcome reporting with this relatively new therapeutic option.

## Data Availability

The anonymized data generated/analyzed during the outlined report may be available from the corresponding author upon request.

## Abbreviations

ADA: Adenosine deaminase 1
Ado: Adenosine
dAdo: deoxyadenosine
CPM: counts per minute
CID: Combined immunodeficiency
dAXP: Total deoxyadenosine nucleotides
ERT: Enzyme replacement therapy
FDA: United States Food and Drug Administration
HSCT: Hematopoietic stem cell transplant
NBS: Newborn screen
PEGylated: Polyethylene glycol-modified
PHA: Phytohemagglutinin
SCID: Severe combined immunodeficiency
SCIG: Subcutaneous immunoglobulin
